# Structural and Functional Small Fiber Abnormalities in the Neuropathic Postural Tachycardia Syndrome

**DOI:** 10.1371/journal.pone.0084716

**Published:** 2013-12-27

**Authors:** Christopher H. Gibbons, Istvan Bonyhay, Adam Benson, Ningshan Wang, Roy Freeman

**Affiliations:** Department of Neurology, Beth Israel Deaconess Medical Center, Harvard Medical School, Boston, Massachusetts, United States of America; University of Iowa Carver College of Medicine, United States of America

## Abstract

**Objective:**

To define the neuropathology, clinical phenotype, autonomic physiology and differentiating features in individuals with neuropathic and non-neuropathic postural tachycardia syndrome (POTS).

**Methods:**

Twenty-four subjects with POTS and 10 healthy control subjects had skin biopsy analysis of intra-epidermal nerve fiber density (IENFD), quantitative sensory testing (QST) and autonomic testing. Subjects completed quality of life, fatigue and disability questionnaires. Subjects were divided into neuropathic and non-neuropathic POTS, defined by abnormal IENFD and abnormal small fiber and sudomotor function.

**Results:**

Nine of 24 subjects had neuropathic POTS and had significantly lower resting and tilted heart rates; reduced parasympathetic function; and lower phase 4 valsalva maneuver overshoot compared with those with non-neuropathic POTS (P<0.05). Neuropathic POTS subjects also had less anxiety and depression and greater overall self-perceived health-related quality of life scores than non-neuropathic POTS subjects. A sub-group of POTS patients (cholinergic POTS) had abnormal proximal sudomotor function and symptoms that suggest gastrointestinal and genitourinary parasympathetic nervous system dysfunction.

**Conclusions and Relevance:**

POTS subtypes may be distinguished using small fiber and autonomic structural and functional criteria. Patients with non-neuropathic POTS have greater anxiety, greater depression and lower health-related quality of life scores compared to those with neuropathic POTS. These findings suggest different pathophysiological processes underlie the postural tachycardia in neuropathic and non-neuropathic POTS patients. The findings have implications for the therapeutic interventions to treat this disorder.

## Introduction

The postural orthostatic tachycardia syndrome (POTS) is defined by an excessive increase in heart rate in the upright position with associated symptoms of orthostatic intolerance [1]. The symptoms of orthostatic intolerance include light-headedness, palpitations, visual blurring, weakness, pre-syncope or syncope when standing. POTS is one of the most prevalent autonomic disorders; current estimates suggest up to 500,000 patients may be affected by the disorder in the United States alone [2].

Heterogeneous pathologies underlie POTS. Several lines of evidence point to a restricted peripheral neuropathy, specifically, sympathetic denervation predominantly in the lower hemibody, as a cause of POTS in some individuals. The evidence includes reports of venous denervation [3], impaired distal sudomotor function [4,5], lower norepinephrine spillover in the legs than the arms [6], and the association with denervation on skin biopsy [7]. This condition is known as neuropathic POTS. However, not all POTS patients have evidence of a peripheral neuropathy. Proposed pathogenic etiologies for non-neuropathic POTS include deconditioning, hypovolemia, low grade inflammation, oxidative stress and genetic causes [5]. In tertiary referral center studies, a neuropathic etiology underlies POTS in 33 to 50% of individuals [6,8]. 

The clinical and neurophysiological differences between neuropathic and non-neuropathic POTS are not fully elucidated. The objective of this study was to test the hypothesis that the features of neuropathic POTS are distinguishable from non-neuropathic POTS when assessed by examination, comprehensive neurophysiologic testing, neuropathology and detailed symptom and functional questionnaires. 

## Methods

### Subjects

Twenty-four patients with POTS and 10 age-matched healthy control subjects were recruited from January 2010-June 2012 for this case-control study. POTS was defined as an increase in heart rate of >30 beats per minute upon standing with symptoms of orthostatic intolerance, without any known medical condition or medication causing the tachycardia. Screening included, but was not limited to, diabetes, impaired glucose tolerance, vitamin deficiencies, heavily metal toxicity, thyroid disorders, pheochromocytoma, hypoadrenalism, anxiety, cardiac disease, volume depletion, drug abuse and medication side effect. Controls were of similar age and gender.

### Protocol approvals, registrations and patient consents

Procedures were approved by the Beth Israel Deaconess Medical Center Institutional Review Board. Written informed consent was obtained from all participants prior to study participation.

### Test Protocol

Individuals participated in 2 testing days separated by no more than 1 month. On day 1, subjects completed questionnaires, had skin biopsies, quantitative direct and indirect sudomotor testing (QDIRT) and quantitative sensory testing (QST). On day 2, subjects underwent autonomic tests that included heart rate variability to deep breathing, blood pressure and heart rate response to a Valsalva maneuver and 10-minute tilt-table test. 

### Neurophysiologic and autonomic testing

*Quantitative direct and indirect reflex testing* was assessed by iontophoresing acetylcholine solution (10%) into the skin of the dorsum of the right foot and the distal right thigh using our previously reported technique [9]. The sweat droplet number and area for both the direct and axon reflex regions were analyzed at 7-minutes post-iontophoresis. Subjects with sweat droplet numbers and/or sweat droplet area below the 5^th^ percentile of the normative range at the foot or thigh at 7-minutes were considered abnormal.

### Quantitative sensory testing

Was performed on the dorsum of the foot and included heat, cold and heat-pain perception thresholds. Testing was performed with ascending (heat and heat-pain) or descending (cold) methods of limits using standard protocols [10]. Thermal stimuli were applied using a 16x16 mm thermode (Medoc TSA II Neurosensory Analyzer). Patients with at least 2 sensory thresholds below the 5^th^ percentile for our laboratory were reported as having an abnormal QST (i.e., individuals with a single abnormal test were still considered normal). 

### Autonomic testing

Subjects rested in a supine position for 20 minutes prior to testing. Blood pressures were measured non-invasively with an automated sphygmomanometer (Colin PressMate, Colin Medical, San Antonio, TX.). Continuous beat-to-beat finger cuff BP recordings were obtained from the left hand (Finapres, Ohmeda, Englewood, CO.). Heart rate variability to deep breathing, expiratory to inspiratory ratio and Valsalva maneuver was measured as previously described [11]. The systolic blood pressure fall during phase II and increase in phase IV (“the overshoot”) of the maneuver were calculated from the beat-to-beat BP measurements throughout the maneuver [12]. A 60° head-up tilt was performed for 10 minutes. 

### Skin biopsy

Subjects had 3mm punch skin biopsies performed at the right leg 10 cm above the lateral malleolus and the right lateral thigh 10 cm above the knee using standard techniques [13]. Specimens were fixed and stained with protein gene product 9.5 (1:1000, rabbit anti-PGP 9.5, Chemicon International Inc.) as previously described [14]. Intra-epidermal nerve fiber density (IENFD) was calculated for each biopsy in a blinded fashion using standard techniques on a florescent microscope (Zeiss-Axioplan2, Germany) [13,15]. The presence of reduced intra-epidermal nerve fiber density at the distal leg (<7 fibers per mm – the 5% normative value cut-off value for immunoflorescent imaging in our laboratory) was considered abnormal.

### Questionnaires

Participants completed the following questionnaires on the first day of the study: the 14-point Chalder Fatigue Scale (CFS) [16], Hospital Anxiety and Depression Scale [17], Krupps Fatigue Severity Scale [18], EuroQol-5D (sub-dimensions scored as normal or abnormal) [19], Orthostatic Intolerance Symptom Assessment [20], Orthostatic Intolerance Disability Assessment [20] and the Boston Autonomic Symptom Questionnaire [21].

### Definition of neuropathic POTS

Neuropathic POTS was defined as a skin biopsy below the 5^th^ percentile, *and* at least 1 other test (quantitative sensory testing or quantitative sudomotor direct and indirect testing) result below the 5^th^ percentile. All other subjects were considered ‘non-neuropathic’.

### Comparison of POTS subjects with and without sudomotor dysfunction

In an additional post-hoc analysis, POTS subjects with sudomotor dysfunction (defined by QDIRT below the 5^th^ percentile at one or more sites) were compared to those with normal QDIRT function at both sites and healthy control subjects.

### Statistical Analysis

Statistical analysis was performed using SPSS 17.0 (IBM Inc. Chicago IL, USA). To avoid bias, all data were de-identified and analyzed in a blinded fashion. Data are reported as means ± standard deviation. Baseline demographic data are analyzed by one-way ANOVA if normally distributed. Neurophysiologic and neuropathologic outcomes are reported using one-way ANOVA with Tamhanes T2 post-hoc tests. Gender distribution and EuroQol questionnaire responses are analyzed by Fisher’s exact test. Other ordinal questionnaire data are analyzed by Kruskal-Wallis test and with Mann-Whitney pairwise testing using Bonferroni correction. Relationships between tests are noted by Pearson correlation coefficient. Significance for all studies is set at *p*<0.05.

## Results

### Demographics

**Figure 1:**
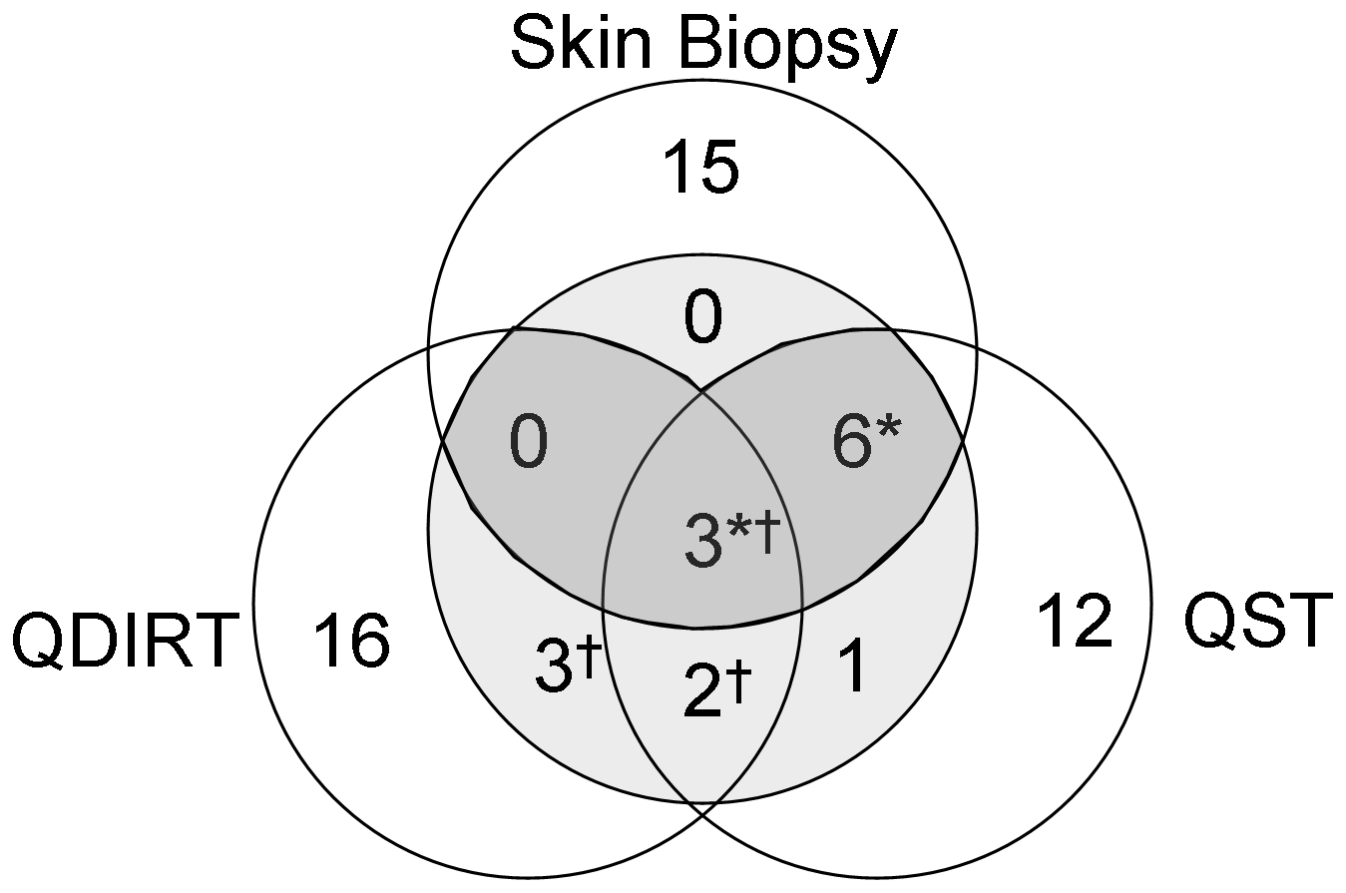
Distribution of test results. Abnormal results are displayed within the lightly shaded region. The 9 individuals that meet criteria for neuropathic POTS (abnormal skin biopsy and 1 other abnormal test) are in the darkly shaded region. A total of 9 subjects had abnormal intra-epidermal nerve fiber density (skin biopsy). A total of 12 individuals had abnormal quantitative sensory testing (QST). A total of 8 individuals had abnormal sudomotor (QDIRT) testing. * = individuals with neuropathic POTS, ^†^ = individuals with cholinergic POTS.

Twenty-four individuals with POTS (22 female) and 10 healthy subjects (7 female) participated in the study. The distribution of defining test abnormalities is shown in Figure 1. Nine patients had *neuropathic* POTS. The remaining 15 patients had *non-neuropathic* POTS. A total of 8 of 24 individuals had sudomotor dysfunction based on QDIRT abnormalities (Figure 1).

The subjects with neuropathic POTS were slightly heavier and had a greater body mass index than individuals with non-neuropathic POTS (Table 1). There were no significant differences in height or age between groups. All male POTS subjects (N=2) had neuropathic POTS. There were no differences in demographic characteristics between individuals with normal and abnormal sudomotor function (Table 2).

**Table 1:** Results for controls, neuropathic and non-neuropathic POTS subjects.

	**Control (10)**	**Non-Neuropathic POTS (15)**	**Neuropathic POTS (9)**
Age (years)	28±7	29±8	34±10
Height (cm)	169±7	166±5	166±13
Weight (kg)	73±14	64±6*	75±14†
Body Mass Index	25.4±3.9	23.2±2.7	27.4±4.0†
Gender	3 M	0 M	2 M
POTS Duration (years)	N/A	4.9±4.4	4.4±3.5
IENFD distal leg (fibers/mm)	14.1±5.5	13.3±3.8	5.1±0.9*†
IENFD distal thigh (fibers/mm)	16.3±5.9	16.8±4.1	8.2±1.1*†
Cold detection (°C)	29.6±1.6	28.6±2.5	29.5±1.2
Heat detection (°C)	36.1±2.81	35.3±2.8	37.9±2.4*†
Heat-pain detection (°C)	45.2±3.3	43.5±4.0	48.3±1.0*†
QDIRT number foot	63±26	54±25	49±32
QDIRT area foot	65±29	48±21	47±32
QDIRT number thigh	39±23	19±10*	30±22†
QDIRT area thigh	41±22	16±8.5*	29±25†
Exp. to Insp ratio	1.49±0.23	1.50±0.16	1.35±0.12
Max-Min heart rate (beats/min)	23.7±11.5	28.3±7.4	19.8±5.4†
Resting heart rate (beats/min)	59±7	71±9*	66±13
Tilt heart rate (beats/min)	89±13	124±10*	114±7†*
Supine SBP	122±15	119±11	121±10
Tilt SBP	118±13	123±13	118±11
Valsalva ratio	1.75±0.19	2.31±0.45*	1.83±0.34†
Valsalva baseline SBP (mmHg)	119±16	121±14	120±13
Valsalva phase 2 early blood pressure (mmHg)	107±13	108±15	108±10
Valsalva phase 2 late blood pressure (mmHg)	121±18	120±26	124±27
Valsalva phase 4 blood pressure (mmHg)	147±18	168±27	132±17*†

Differences between groups measured by ANOVA with Tamhanes T2 post-hoc test (except gender, Fishers exact test). *= P<0.05 vs. Control. †= P<0.05 neuropathic vs. non-neuropathic. SBP: systolic blood pressure. Exp. to Insp ratio- the expiratory to inspiratory ratio during deep breathing.

**Table 2:** Results in individuals with normal and abnormal sudomotor function.

	**Control (10)**	**Normal sudomotor function (16)**	**Abnormal sudomotor function (8)**
Age (years)	28±7	29±9	35±9
Height (cm)	169±7	167±9	162±7
Weight (kg)	73±14	68±12	67±8
Body Mass Index	25.4±3.9	24.3±3.9	25.7±3.8
Gender	3 M	2 M	0 M
POTS Duration (years)	N/A	5.4±4.1	3.4±3.7
IENFD distal leg (fibers/mm)	14.1±5.5	9.8±4.6	10.9±6.2
IENFD distal thigh (fibers/mm)	16.3±5.9	13.6±5.5	13.5±5.4
Cold detection (°C)	29.3±1.6	29.5±1.3	27.8±3.1
Heat detection (°C)	36.1±2.81	35.6±2.5	37.1±3.5
Heat-pain detection (°C)	45.2±3.3	45.0±3.7	45.9±4.8
QDIRT number foot	63±26	62±23	34±28
QDIRT area foot	65±29	56±21	33±28
QDIRT number thigh	39±23	29±16	12±8
QDIRT area thigh	41±22	26±18	12±8
Exp:Inspiratory ratio	1.49±0.23	1.46±0.17	1.43±0.15
Max-Min heart rate (beats/min)	23.7±11.5	26±8	23±7
Resting heart rate (beats/min)	60±7	70±11	69±12
Tilt heart rate (beats/min)	89±13	121±12	120±8
Supine SBP	122±15	120±12	122±11
Tilt SBP	118±13	120±11	119±12
Valsalva ratio	1.75±0.19	2.21±0.51	1.98±0.39

SBP: systolic blood pressure. Exp. to Insp ratio- the expiratory to inspiratory ratio during deep breathing.Intra-epidermal nerve fiber density

Subjects with neuropathic POTS (per definition) had lower IENFD than the non-neuropathic POTS group and controls (*p*<0.05 ANOVA, both sites, Table 1). There were no IENFD differences between controls and non-neuropathic POTS subjects. 

### Quantitative sensory testing

There were significantly higher heat and heat-pain detection thresholds in the neuropathic POTS group compared to the non-neuropathic and control subjects (*p*<0.05 ANOVA, Table 1). There were no differences in QST results between the non-neuropathic POTS group and the controls. There were no differences in cold detection thresholds between groups. 

### Autonomic testing

Resting and upright heart rate was higher in POTS subjects than controls. Subjects with neuropathic POTS had lower resting and upright heart rates than non-neuropathic POTS subjects (Table 1). Heart rate variability to deep breathing and Valsalva maneuver was lower in neuropathic compared to non-neuropathic POTS subjects (table 1). Baseline blood pressures and phase 2 blood pressure responses were similar in all groups. The Valsalva phase 4 overshoot was lower in neuropathic POTS patients than controls and non-neuropathic POTS. Subjects with non-neuropathic POTS had greater Valsalva phase 4 overshoot than control subjects. 

### Quantitative direct and indirect reflex testing of sudomotor function (QDIRT)

There were no differences in sweat droplet number or area between subjects with neuropathic POTS, non-neuropathic POTS and control subjects at the distal leg. There was lower sweat area and droplet number in the axon-reflex region of the distal thigh in subjects with non-neuropathic POTS (table 1). 

### Correlations between tests

There were strong correlations between tests of autonomic function and the nerve fiber density at the distal leg. In all subjects (POTS and controls), the IENFD at the distal leg correlated with the expiratory to inspiratory ratio (r=0.61, P<0.001) and heart rate variability to deep breathing (r=0.55, P<0.01). In POTS subjects, IENFD at the distal leg correlated with the expiratory to inspiratory ratio (r=0.55, P<0.05), heart rate variability to deep breathing (r=0.63, P<0.01), the Valsalva ratio (r=0.46, P<0.05) and the Valsalva phase 4 blood pressure overshoot (r=0.46, P<0.05). 

### Questionnaires

**Figure 2:**
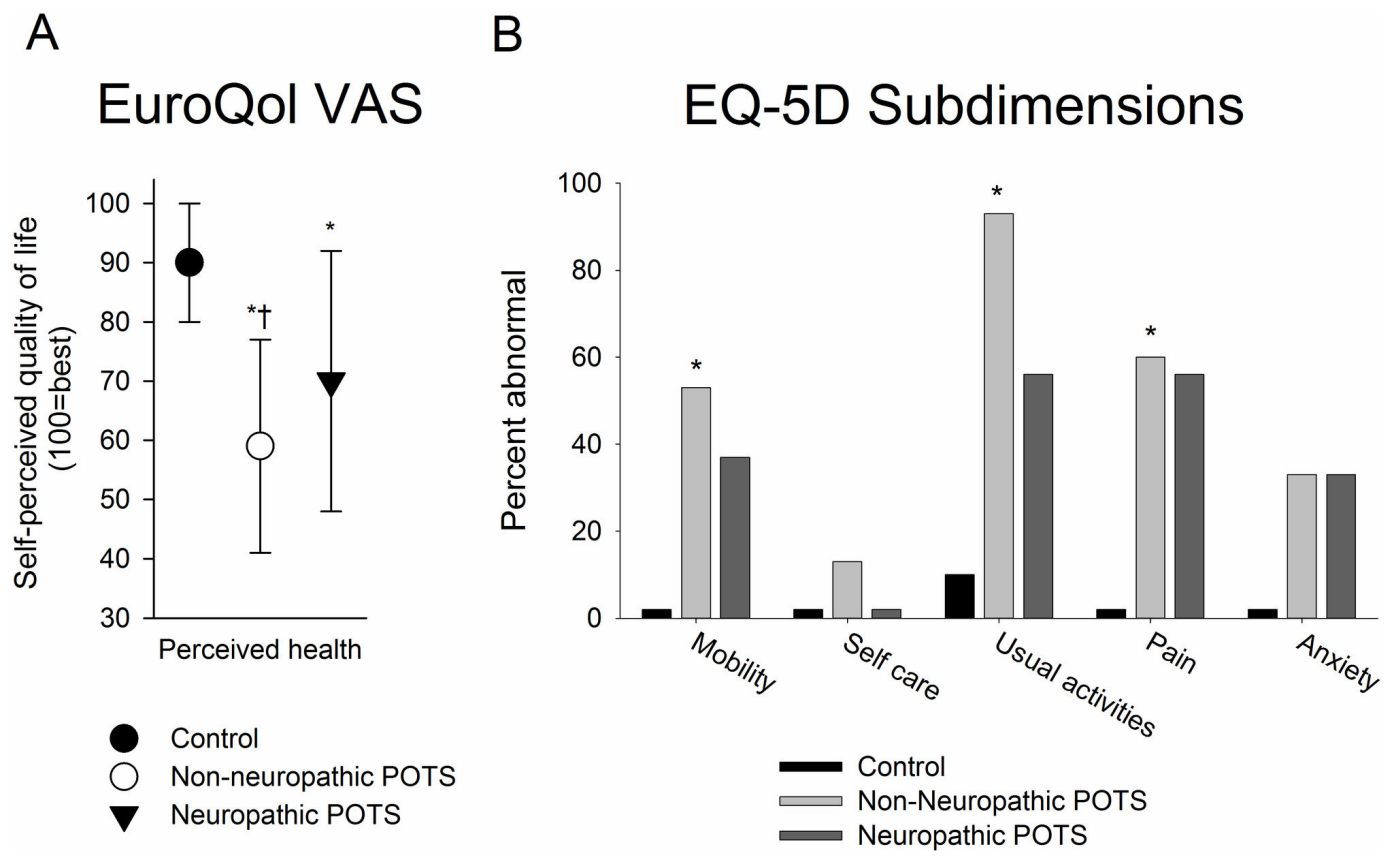
Results of European Quality of Life Questionnaire. A. The EuroQol VAS for self-perceived healthy-related quality of life (Score of 100 = best imaginable health state, 0= worst imaginable health state). The non-neuropathic POTS had the lowest perceived quality of life, while the neuropathic POTS subjects had lower self-perceived health than control subjects. * *p*< 0.01 vs. controls, † *p*<0.01 vs. neuropathic POTS (ANOVA with Tamhanes T2 post-hoc test). The EuroQol 5D (B) shows the individual sub-dimensions with the percent of individuals reporting abnormalities shown. Significant differences in mobility, usual activities and pain were noted between groups (*=P<0.05, Fishers exact test). A greater percent of non-neuropathic POTS subjects reported self-perceived difficulty across all sub-dimensions than control and neuropathic POTS subjects (with the exception of anxiety which was reported with the same frequency as neuropathic POTS subjects).

All questionnaire results are reported in Table 3 and Figure 2. High levels of fatigue and orthostatic intolerance were seen in patients with neuropathic and non-neuropathic POTS on the Chalder and Krupps fatigue scales, and the orthostatic intolerance scales. On the Hospital Anxiety and Depression Scale, subjects with non-neuropathic POTS endorsed significantly more symptoms of depression and anxiety compared to control subjects. Individuals with neuropathic POTS were not significantly different from controls. On the EuroQOL visual analogue scale, individuals with non-neuropathic POTS reported significantly lower self-perceived overall health-related quality of life than neuropathic POTS patients and control subjects. Among the EuroQoL-5D sub-dimensions, mobility, ability to carry out usual activities, and presence of pain or discomfort were greatest in non-neuropathic POTS subjects (Figure 2). 

**Table 3:** Questionnaire Data.

**Questionnaire**	**Control (10)**	**Non-neuropathic POTS (15)**	**Neuropathic POTS (9)**
Chalder fatigue scale	12.5±3.7	24.2±7.2*	22.2±9.4*
Chalder physical	7.0±2.6	15.4±4.5*	14.3±6.3*
Chalder mental	5.5±1.4	9.0±3.1*	8.0±3.3*
Krupps fatigue scale	2.0±0.4	4.8±1.2*	4.1±1.5*
Orthostatic Intolerance Symptom Assessment	1.3±1.9	28.5±14.7*	29.3±12.6*
Orthostatic Intolerance Disability Assessment Scale	0.5±0.8	14.3±9.6*	15.7±11.9*
Hospital Anxiety and Depression Scale (HADS)	3.4±2.7	11.9±5.3*	9.7±8.1
HADS anxiety	1.5±1.3	5.5±2.3*	4.6±4.4
HADS depression	1.8±1.6	7.5±3.6*	5.1±4.1

Differences between groups measured by Kruskal-Wallis test and with Mann-Whitney pairwise testing using Bonferroni correction to define differences between groups. *= Significant difference vs. Control.

Symptoms of orthostatic intolerance and orthostatic disability were similar in the neuropathic and non-neuropathic POTS groups and significantly more than in the controls (Table 1). There were no significant differences among neuropathic POTS and non-neuropathic POTS groups in gastrointestinal, genitourinary, cardiovascular and vasomotor, autonomic symptoms using the Boston Autonomic Symptom Questionnaire. 

### Sudomotor function analysis

IENFD, QST and cardiovascular autonomic function was similar in individuals with and without sudomotor dysfunction (table 2). Individuals with sudomotor dysfunction reported greater difficulty with urinary flow (2.8±3.6 vs. 0.2±0.6; *p*<0.05), greater incontinence (6.1±4.0 vs. 2.7±3.6; *p*<0.05), early satiety (0.7±0.9 vs. 0.1±0.2; *p*<0.05) with more nausea and vomiting (4.4±3.2 vs. 1.9±1.6; *p*<0.05) than individuals with normal sudomotor function.

## Discussion

In this manuscript, we report the first comprehensive analysis of the symptoms, neurophysiology and cutaneous neuropathology of individuals with neuropathic and non-neuropathic POTS. The major findings are: compared to subjects with non-neuropathic POTS, subjects with neuropathic POTS have 1) lower resting and tilted heart rates; 2) lower measures of parasympathetic function; 3) lower phase 4 Valsalva maneuver overshoot; 4) lower anxiety and depression scores; 5) greater mobility, and greater ability to carry out usual activities; and 6) greater self-perceived overall health related quality of life. In addition, we report a sub-group of POTS patients with abnormal proximal sudomotor function and symptoms that suggest gastrointestinal and genitourinary parasympathetic nervous system dysfunction. 

There are no widely accepted definitions or diagnostic criteria for neuropathic POTS. Previously used definitions include those based on impaired cardiovascular autonomic function [5], impaired sudomotor function [8], and impaired distal norepinephrine release [6]. Consistent with the notion that neuropathic POTS represents a distal autonomic and small fiber neuropathy, our diagnostic criteria included distal small fiber pathology, impaired distal sensory function and impaired distal sudomotor function. We included impaired sudomotor function in this definition because this is the most distal autonomic function measure but excluded measures of heart rate variability because these are more proximal measures and seemed likely to be impaired only in more severe cases of POTS. Our definition thus has a structural small fiber criterion (intra-epidermal nerve fiber density), a functional small fiber criterion (quantitative sensory testing) and a functional autonomic criterion (quantitative sudomotor function testing). The differences in cardiac vagal function between the neuropathic and non-neuropathic POTS groups and the strong correlations between cardiac vagal function measures and intra-epidermal nerve fiber density lends support to this classification.

The phase IV overshoot of the Valsalva maneuver was greater in the non-neuropathic POTS subjects compared to the control and neuropathic POTS subjects. Prior studies in both control [22] and POTS [23] subjects have shown that the increased phase IV overshoot is the result of a hyperadrenergic state. Our data suggest that the hyperadrenergic state is most prominent in the non-neuropathic group. Consistent with this conclusion, the non-neuropathic POTS group had a greater Valsalva ratio compared to control and neuropathic POTS subjects. 

The structural criterion, reduced intra-epidermal nerve fiber density, was abnormal in 38% of POTS subjects. This differs from the study of Singer et al. in which none of 8 POTS subjects had intra-epidermal nerve fiber densities in the abnormal range, although morphological changes in the intra-epidermal nerves were present in 3 of the 8 subjects [7]. In contrast, in that study 7 of the 8 subjects showed reduced sweating measured by QSART, and were similar to our POTS group with sudomotor dysfunction [7].. These data lend support to the lack of concordance between IENFD and sudomotor function testing that was observed in our study and emphasize the heterogeneity of the POTS population, even among those with evidence of neuropathy. Furthermore, our subjects with neuropathic POTS did not report any symptoms of a painful small fiber neuropathy. Although a number of disorders are now associated with asymptomatic small fiber neuropathy [24], the pathophysiologic differences between individuals with and without symptoms of a small fiber neuropathy has not been elucidated. The heterogeneity among subjects suggests different pathophysiological mechanisms underlie the clinical features of the disorder. The data suggest that even in those patients in whom an immunological cause underlies the POTS, different target nerve epitopes are likely to be implicated and the response to therapeutic interventions is likely to differ among phenotypes. This may underlie the challenges that exist in treating patients with POTS. 

The POTS cohort had mean fatigue levels that were significantly different to control subjects and approached those of chronic fatigue syndrome patients. These data confirm the well-established overlap between the POTS and chronic fatigue syndrome [25-27]. A recent study drew attention to the presence of deconditioning in POTS patients [28]. While the present data do not help disentangle the interaction between deconditioning and POTS, the presence of these levels of fatigue provides a possible explanation for the observed deconditioning and suggests that deconditioning may be present irrespective of POTS etiology. These data, in part, may underlie our preliminary observation that symptoms of POTS persist despite a rigorous reconditioning exercise program [29].

Anxiety and depression were greater in POTS patients than controls. Furthermore, both anxiety and depression were greatest in non-neuropathic POTS. Similarly, overall perceived health status and the sub-dimensions of mobility and usual activities tended to be worse in non-neuropathic POTS patients. Irrespective of etiology, the perceived health status scores relative to controls and neuropathic POTS patients suggest that reduced physical activity and deconditioning is likely to be greater in this sub-group and implementation of a reconditioning exercise program harder.

All non-neuropathic POTS subjects were females whereas, 22% of the neuropathic POTS group were males. Several studies have drawn attention to the greater predisposition to orthostatic intolerance in females compared to males [30-32]. The physiological factors that underlie this predisposition include decreased stroke volume [33,34], decreased total muscle sympathetic outflow [35] and differences in sympathetic burst discharge characteristics [36]. The present study suggests that the gender predisposition to increased orthostatic intolerance may be particularly applicable to the non-neuropathic POTS subgroup. 

Axon-mediated sudomotor test abnormalities occurred in 33% of our POTS cohort. The prevalence of sudomotor abnormalities is slightly lower than prior reports in which axon-reflex mediated sudomotor impairment was present in 43-63% of subjects [5,8,37]. The relative difference in proportions of the POTS subgroups most likely reflects the referral biases of the respective centers.

The sudomotor abnormalities in our POTS cohort did not have a distal to proximal gradient. Similar observations have been made by other investigators studying thermoregulatory and axon-mediated sweating in POTS. Thieben et al. observed a regional or mixed pattern in 20% of patients [8], Peltier et al. reported patchy sudomotor abnormalities [37] while Khurana observed predominantly upper limb anhidrosis in 50% of subjects [38]. 

Sudomotor abnormalities in our cohort occurred less frequently than intra-epidermal nerve fiber density abnormalities and QST abnormalities. In contrast, QSART dysfunction was the most common abnormality in the autonomic neuropathy cohort of Sheklee et al. occurring in 63% of subjects [5]. In that cohort, gastrointestinal symptoms such as abdominal pain, bloating, nausea and constipation occurred in almost 70% of subjects. Their cohort has features in common with our POTS subjects with sudomotor dysfunction in which gastrointestinal symptoms such as early satiety, nausea and vomiting occurred. In our POTS subjects with sudomotor dysfunction, urinary flow and urinary incontinence also occurred more frequently in this POTS subgroup raising the possibility of a generalized dysfunction of the cholinergic system. 

These POTS patients with sudomotor dysfunction bear some similarity to post-ganglionic cholinergic dysautonomia which is characterized by generalized, post-ganglionic parasympathetic nervous system impairment that includes gastrointestinal and bladder dysfunction, pupillomotor deficits, sudomotor dysfunction, xerophthalmia and xerostomia [39-43]. Compared to reported cases of post-ganglionic cholinergic dysautonomia, the parasympathetic impairment in this POTS subgroup was, as expected, less prominent. But the prevalence in our series is similar to previously reported series on idiopathic autonomic neuropathy in which 20%[44] to 26%[45] of subjects presented with pure cholinergic dysautonomia. 

The cholinergic POTS subgroup is therefore consistent with the hypothesis that neuropathic POTS is a restricted autonomic neuropathy [6]. Post-ganglionic cholinergic dysautonomia is frequently associated with a viral infection and is most likely immune-mediated [6,39-45]. The absence of a distal to proximal gradient in sudomotor deficits, observed by us and by others lends additional support to the possibility that an immune-mediated process underlies neuropathic POTS [5,8,37]. Taken together, these data provide support for heterogeneity even among neuropathic POTS subgroups. 

The cause of the exaggerated postural tachycardia in the cholinergic dysautonomic group is not immediately apparent. While impaired cardiac vagal function could provide a possible pathophysiological explanation, heart rate variability with deep respiration and the heart rate response to a Valsalva maneuver, measures of cardiac vagal function, were similar to the control subjects in this group. Alternatively, there could be impaired selective distal vasomotor denervation but this was not evaluated in this study. 

There are limitations to our study. The POTS patients were recruited at a tertiary-care center with a special interest in peripheral neuropathy. The numbers are small and the relative proportions of neuropathic and non-neuropathic POTS patients cannot be generalized to the general population. The concept of cholinergic POTS is based on a post-hoc analysis. This should be examined prospectively in future studies. Nevertheless, the study demonstrates that there are several POTS subgroups with coherent symptoms, and structural and functional features. These findings may have implications for the natural history of POTS and the response of POTS patients to therapeutic interventions. 
